# Texture and High Yield Strength of Rapidly Solidified AZ31 Magnesium Alloy Extruded at 250 °C

**DOI:** 10.3390/ma16082946

**Published:** 2023-04-07

**Authors:** Peiran Ye, Chao Yang, Zhenshuai Li, Shuai Bao, Yuchu Sun, Wucheng Ding, Yungui Chen

**Affiliations:** 1 Institute of New Energy and Low-Carbon Technology, Sichuan University, Chengdu 610207, China; 2 School of Materials Science and Engineering, Sichuan University, Chengdu 610065, China; 3 School of New Energy and Materials, Southwest Petroleum University, Chengdu 610500, China; dwc20150520@163.com; 4 Engineering Research Center of Alternative Energy Materials & Devices, Ministry of Education, Chengdu 610065, China

**Keywords:** hot-extruded AZ31 alloy, rapid solidification powder metallurgy, mechanical property, grain refinement, unusual texture

## Abstract

In this study, commercial AZ31B magnesium alloy was used to compare the differences between the microstructure, texture, and mechanical properties of conventional solidification (as homogenized AZ31) and rapid solidification (as RS AZ31). The results demonstrate that a rapidly solidified microstructure leads to better performance after hot extrusion with a medium extrusion rate (6 m/min) and extrusion temperature (250 °C). The average grain size of as-homogenized AZ31 extruded rod is 100 μm after annealing and 4.6 μm after extrusion, respectively, but that of the as-RS AZ31 extruded rod is only about 5 μm and 1.1 μm, correspondingly. The as-RS AZ31 extruded rod attains a high average yield strength of 289.6 MPa, which is superior to the as-homogenized AZ31 extruded rod, and is improved by 81.3% in comparison. The as-RS AZ31 extruded rod shows a more random crystallographic orientation and has an unconventional weak texture component in <$11\bar{2}1$>/<$20\bar{2}1$> direction, which has not been reported yet, while the as-homogenized AZ31 extruded rod has an expected texture with prismatic <$10\bar{1}0$>/<$\bar{1}2\bar{1}0$>//ED.

## 1. Introduction

In order to reduce carbon emissions, researchers have launched a revolution in lightweight materials to reduce the use of fossil fuels. Magnesium (Mg) alloys are seen as the underlying lightweight structural metals of the coming age, as their weight savings can be efficiently translated into lower energy consumption [1]. However, Mg alloys are generally limited by poor yield strength at room temperature [2] and exhibit a narrow window of plastic formability [3], making it challenging and costly to process profiles or components by using traditional casting and forming techniques, which also hinders their widespread applications.

The AZ31B alloy is one of the commercially available and least expensive Mg alloys and has a limited solid solubility of Al and Zn in the binary system with Mg, which is 11.5 at% and about 2.8 at%, respectively [4]. After casting, the β-Mg_17_Al_12_, which is the dominant second phase of AZ series alloys and has a low melting point of approximately 460 °C and is comparatively soft at lower temperatures, does not serve to pin boundaries [5]. Accordingly, the volume fraction of β-Mg_17_Al_12_ in AZ31 alloy is too small to be considered to have good strengthening during hot deformation [6,7]. However, in order to further improve the strength of AZ31, according to the Hall-Petch relationship, the most effective method is to reduce the grain size of the alloy [8].

The extrusion process is a primary forming step to refine the microstructure, which is usually worked at an elevated temperature for Mg alloys [9]. Since high temperatures can activate the non-basal slip, this is beneficial for enhancing the plastic formability of Mg alloys [10]. Previous hot extrusion-related research of AZ31 alloy showed that: (i) considering the operation continuity, productivity, and so on, the extrusion speed range for basic profiles was about 20~30 m/min, but the extrusion temperature was at least greater than 350 °C [9,11]; (ii) the tensile yield strength of AZ31 extruded rods was less than 200 Mpa, and the average grain size of them was more than 5 μm [12,13,14]; (iii) AZ31 extruded rods also had a fiber texture with <$10\bar{1}0$> or {$11\bar{2}0$} component parallel to the extrusion direction [15,16,17]. In other words, the AZ31 extruded alloy needs to be processed at a relatively high temperature to meet the available productivity, but the higher the temperature, the greater the grain size, the poorer the performance, and the more severe the texture [18,19].

Therefore, the present researchers have focused on lower temperatures, seeking a better understanding of the performance of the AZ31 alloy. Previous studies showed rapid solidification (RS) is a type of non-equilibrium solidification process, and its solidification rateis much higher than that of conventional casting (generally referring to the cooling rate of more than 10^5^~10^6^ K/s) [20,21], which will significantly refine the initial microstructure for Mg alloys. Lee et al. [22] used rapidly solidified flaky powder metallurgy (RS FP/M) to obtain AZ91 alloy with uniformly distributed second-phase particles and an ultra-fine homogeneous microstructure whose average grain size was 1.1 μm, and compared with the same cast extrusion process, the yield strength increased by 134 Mpa. Chen et al. [23] studied that the RS AZ31 ribbons improved the yield strength by 140 Mpa with grain refinement compared with AZ31 ingot after extrusion at 300 °C. After rapid solidification, it is found that Mg alloys can form sub-micro- or nano-scale microstructures, improve the supersaturation of alloying elements in the matrix, and inhibit the formation of intermetallic compounds, which naturally improve the strength and plasticity of Mg alloys [24,25,26]. However, deforming at excessively high temperatures will destroy the advantage of the initial fine RS microstructure. Ayman et al. [27] demonstrated the RS P/M Mg alloy extruded at 300 °C had a higher Zener–Hollomon parameter (Z values) than that extruded at 400 °C, indicating that it leads to a weaker texture and reduced anisotropy. In addition, comparing the mechanical properties and microstructure after extrusion at 260~400 °C, the finest grain sizes of RS Mg alloys were achieved at 260 °C, thereby achieving the highest yield stress values [28]. 

At the same time, the application of severe plastic deformation (SPD) techniques leading to the formation of ultrafine-grained (UFG) microstructure, for example, equal channel angular pressing (ECAP), extrusion of recycled chips, etc., is found to be beneficial for improving mechanical properties. Some researchers used techniques such as high-ratio differential speed rolling and multi-pass equal channel angular extrusion to achieve high yield strengths for AZ31 alloys with 370~380 Mpa processed at 150~200 °C. Nevertheless, it is difficult to mass manufacture by using the SPD technique because it relies on a very low strain rate (leading to low productivity) and is usually accompanied by extreme basal texture and symmetric anisotropy (leading to non-uniform performance) [29,30].

Hence, rapid solidification can be considered one of the effective ways to refine magnesium alloy grains to improve yield strength. In general, the small average grain size and overall weak texture, caused by the random orientation of small DRX grains, resulted in relatively high yield strength and moderate elongation during both tensile and compressive loading. Nevertheless, previous studies have not clearly shown the differences in microstructure between rapid solidification and conventional solidification. The potential effects of the initial rapidly solidified microstructure and texture on the deformation process also have not been fully investigated. In the current work, the ultra-fine microstructure and superior tensile yield strength of hot-extruded AZ31 rod were obtained by a rapid solidification and extrusion process with a medium extrusion speed and extrusion temperature. So, this work goes on analyzing why the rapidly solidified microstructure would lead to differences in the grain size, texture, and mechanical properties of AZ31 extruded rod. Moreover, it can open the way to the industrial application of RS process in the development of high-performance Mg alloy products.

## 2. Experimental

In the first commercial AZ31B Mg alloy purchased from the Shenzhen Rui Hong Jia Metal Material Co. for remelting, one part was homogenized and then extruded, and the other part was rapidly solidified, pulverized, ingot pressed, and extruded at last. According to Murai et al. [31], the homogenization treatment used annealing at 400 °C for 10 h and cooling to room temperature in air to eliminate Mg-Al and Mg-Al-Zn eutectic phases in remelted AZ31B alloy. Then, the RS process used the single-roller melt-spinning method. The AZ31B was remelted at 730 °C in a mixed protective atmosphere of CO_2_ and SF_6_. The single roller was in the CO_2_ atmosphere, and the linear speed of the roller was controlled at 15 m/s. The RS AZ31 ribbons were ~120 μm thick, ~2.5 mm wide, and ~10 mm long. Next, the RS AZ31 ribbons were pulverized by using a planetary ball mill operating in an Ar atmosphere with a rotational speed of 120 rpm, a ball milling time of 12 h, and a charge ratio of 20:1. Before being stored in aluminum-plastic bags under vacuum, the RS powders were sieved to sizes ranging from 75 to 200 μm using standard sieves (GB 6005–1985). Finally, the RS powders were cold pressed at 400 Mpa for 2 min, then heated to 150 °C and hot pressed at 500 Mpa for 2 min. The AZ31 ingots, after homogenization (as-homogenized) and rapid solidification (as-RS), were extruded at 250 °C with an extrusion speed of 6 m/min and an extrusion ratio of 25:1. The diameter of the hot-extruded rod was 5 mm.

An X-ray diffractometer (XRD, PANalytical Empyrean, Almelo, The Netherlands) was carried out by using CuKa radiation to investigate the physical structure of as-RS and as-homogenized with a scanning range from 30° to 75° and a step size of 0.02°. The samples were polished after grinding with SiC sandpaper, followed by etching with a corrosive solution (1 mL nitric acid + 1 mL acetic acid + 1 g oxalic acid + 150 mL water). Optical microscopy (OM, Model Zeiss Lab A1, Jena, Germany) and scanning electron microscopy (SEM, JSM-6490LV type, Hitachi, Japan) were used to analyze the microstructure of as-RS, as-homogenized, and hot-extruded rods. The high-angle annular dark field scanning transmission electron microscopy (HAADF-STEM) and scanning transmission electron microscopy energy dispersive X-ray spectroscopy (STEM-EDXS) tests were performed in a transmission electron microscope (Talos F200S, Waltham, MA, USA) equipped with a SUPER X EDX detector. The STEM samples were mechanically thinned to 100 μm thickness and then thinned using focused ion beam (FIB; Thermo Scientific Helios 5 CX, Waltham, MA, USA) technique. Grain size, texture, and other relevant microstructural features of the as-RS and as-homogenized AZ31 hot-extruded rods were obtained using electron backscattered diffraction (EBSD, Oxford Nordly max3, Oxford, UK). All the samples for EBSD were electropolished in a solution of 15 mL HclO_4_ and 135 mL alcohol at about −40 °C to −20 °C and a voltage of 4 V for ~3 min. The tensile tests on the dog-bone-shaped specimens with a diameter of 2.5 ± 0.5 mm and a parallel length of 28 mm were performed on a computer-controlled universal testing machine (Instron 5569, Norwood, MA, USA) at room temperature with a tensile strain rate of 2.8 × 10^−4^ s^−1^. In addition, the tensile tests were repeated at least three times for each specimen to reduce the experimental errors.

## 3. Results and Discussion

### 3.1. Microstructure of Conventional and Rapid Solidification

**Figure 1:**
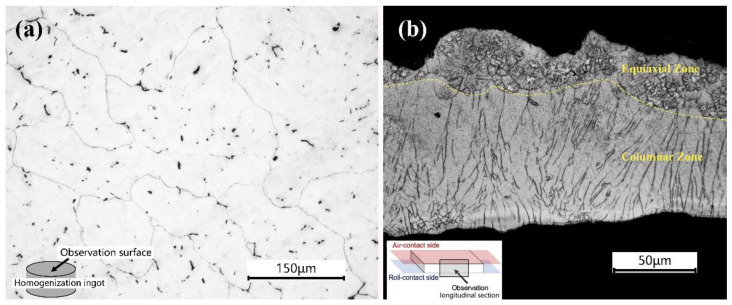
Optical microstructure of (**a**) AZ31 homogenized ingot (as-homogenized) and (**b**) longitudinal section of RS AZ31 ribbons (as-RS).

Figure 1a shows the microstructure of an AZ31 ingot (as-homogenized), and it can be seen that the grain size of the ingot is about 100 μm, and a few second phases are observed. Figure 1b shows the optical micrograph of a longitudinal section of the RS ribbon zone (as-RS) with a thickness of about 100 μm, which can be divided into a columnar zone and an equiaxial zone. The equiaxial zone is the solidification area on the air-contact side, accounting for about 25%, where the internal α-Mg grains are branched, and the size of their secondary dendrites is not much different from that of the grains in the columnar zone, about 5 μm. It is shown that the grains of the as-RS AZ31 are much finer than those of the as-homogenized AZ31.

**Figure 2:**
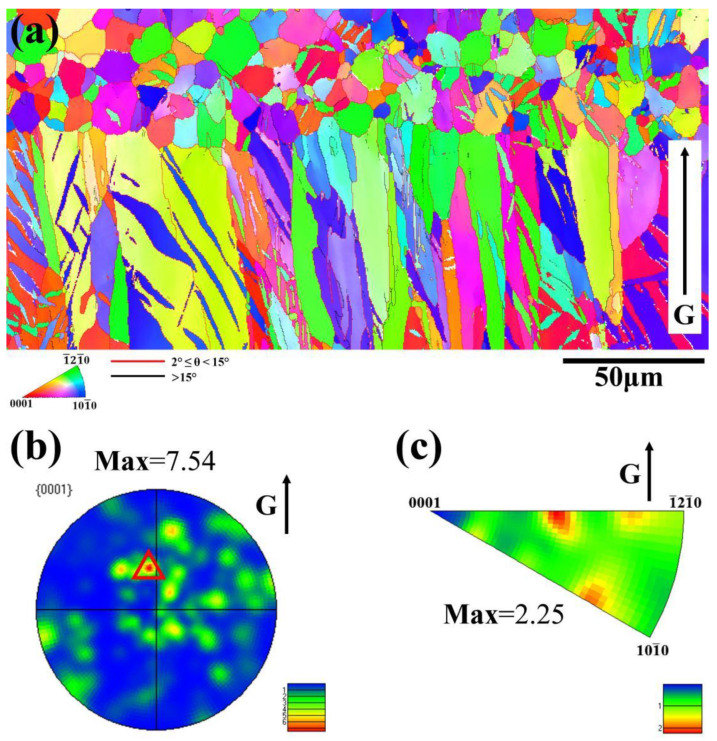
EBSD results of longitudinal section of as-RS AZ31: (**a**) IPF map; (**b**) {0001} PF; (**c**) IPF along thermal gradient.

Figure 2 also shows the EBSD results of the longitudinal section of as-RS AZ31 from Figure 1b. The inverse pole figure map (IPF) presented in Figure 2a more clearly reveals that there is a considerable difference in the microstructure between the columnar zone and the equiaxial zone. It can be obviously seen that the thickness of the equiaxial zone and the columnar zone is approximately 45 μm and 73 μm, respectively, as well as that there is an abundance of low angle grain boundaries (LAGBs, 2° ≤ θ < 15°), indicating that RS AZ31 has a high dislocation density due to competing growth of columnar grains. Figure 2b,c show the texture of as-RS AZ31. The {0001} pole figure (Figure 2b) and corresponding IPF (Figure 2c) show that there is a non-fibrous texture at ND, but the maximum intensity (7.54) in {0001} PF marked as a red triangle indicates that the c-axis directions of most grains are about 30° towards the thermal gradient (G). 

**Figure 3:**
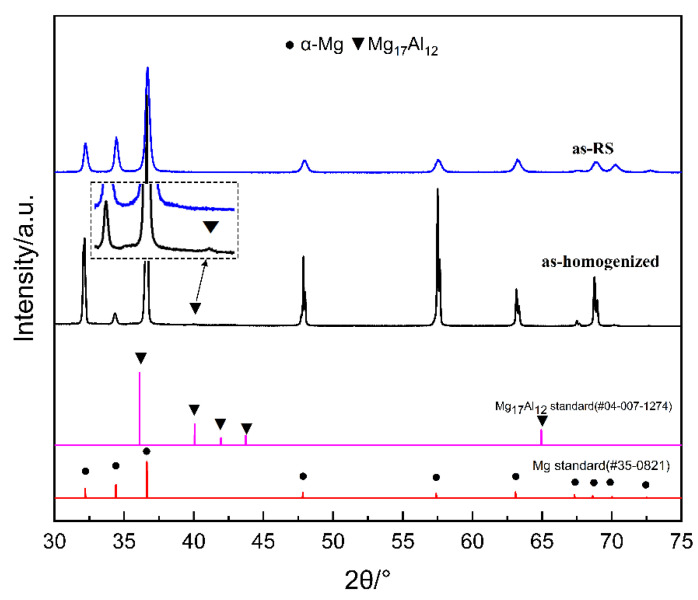
The XRD patterns of as-homogenized and as-RS AZ31B alloys.

Figure 3 shows the XRD results of as-homogenized and as-RS AZ31. The characteristic peaks correspond to the International Center for Diffraction Data (ICDD) PDF card #35-0821 of α-Mg and #04-007-1274 of Mg_17_Al_12_. Obviously, the peaks of Mg_17_Al_12_ are still indexed in the spectra of the as-homogenized AZ31, which is related to the air-cooling after homogenization treatment. However, they are not observed in as-RS AZ31, which is in line with the other RS process reported [32]. In addition, the characteristic peak positions of α-Mg in as-RS AZ31 are shifted by about 0.1° towards the large angle direction compared to the standard Mg (PDF#35-0821), which is the result of alloying elements such as Al and Zn with smaller atomic radii displacing the α-Mg atoms, leading to a reduction in the inter-planar crystal spacing (atomic sizes: Mg/160 pm, Zn/134 pm, Al/143 pm). From the XRD analysis, it can be concluded that the rapid solidification process can improve the solid solution strengthening of the Mg matrix more than the homogenization treatment.

### 3.2. Microstructure of Hot-Extruded Rods

**Figure 4:**
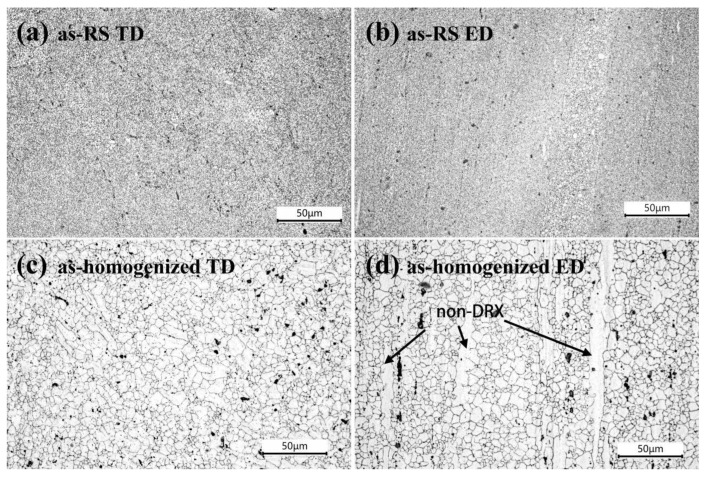
Optical microstructure of the extruded rods of (**a**,**b**) as-RS AZ31 and (**c**,**d**) as-homogenized AZ31.

Figure 4 shows the microstructure of as-RS and as-homogenized AZ31 extruded rods in the extrusion direction (ED) and transverse direction (TD). Figure 4a,b show the microstructure in the direction of as-RS on the TD and ED, respectively. It can be found that the dynamically recrystallized (DRXed) grains are very tiny and uniform in size, about 1 μm. In contrast, the size of the DRXed grains of as-homogenized AZ31 on the TD and ED is uneven, with the largest grain size that can be seen being over 10 μm and the minimum being 1.5 μm, as well as a small amount of non-DRXed grains that can be significantly observed in the ED direction, as seen in Figure 4c,d.

**Figure 5:**
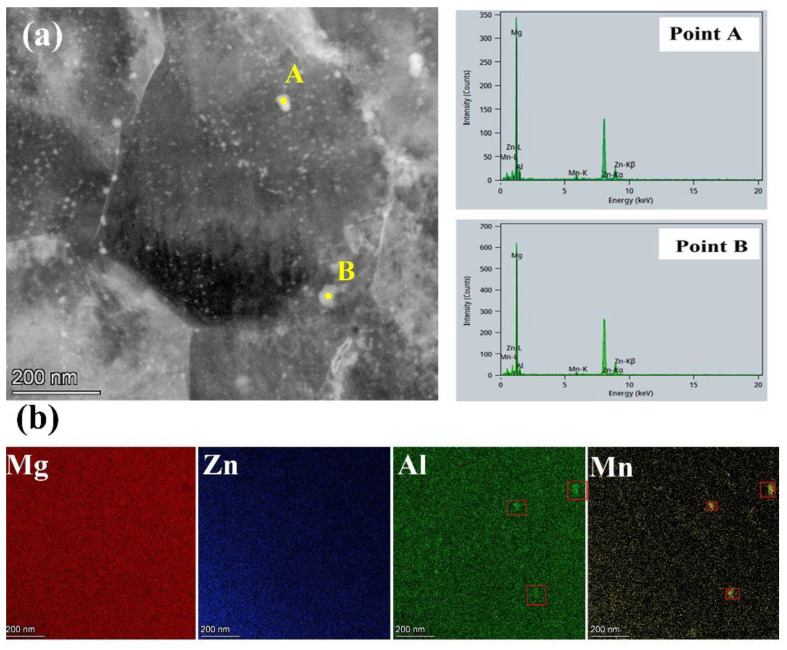
HAADF-STEM (**a**) and entire zone STEM-EDXS mapping (**b**) of as-RS AZ31 extruded rod.

Using HAADF-STEM and EDXS mapping, the finer microstructure and elemental distributions are observed in the as-RS AZ31 extruded rod, as shown in Figure 5. It is found that there are many second-phase precipitates in Figure 5a, whose size is 10~30 nm in diameter and are distributed within the sub-micron grain of 400~600 nm in diameter and grain boundary (GB). The EDXS mapping across the entire zone in Figure 5a shows that Mg and Zn elements are uniformly distributed in α-Mg matrix without obvious segregation in Figure 5b, indicating that rapid solidification can reduce the segregation of solute atoms and expand the solid solubility of Al and Zn, which is conducive to the homogenization of alloying elements. When combined with EDS scanning at points A and B in Figure 5a and the EDXS element mapping in Figure 5b, it is found that Al and Mn aggregate in the nanoparticles, which determines that they are mainly Al-Mn particles. According to relevant studies [33], this Al-Mn phase is almost an Al_8_Mn_5_ phase. These dense nanoparticles observed in the as-RS AZ31 extruded rod will ultimately affect the strength of the alloy [34]. Although nanoparticles have been observed in Figure 5, it has been concluded from EDS mapping that there is no significant and consecutive elemental segregation at the grain boundaries of the sub-micron grains that formed after extrusion of as-RS AZ31.

**Figure 6:**
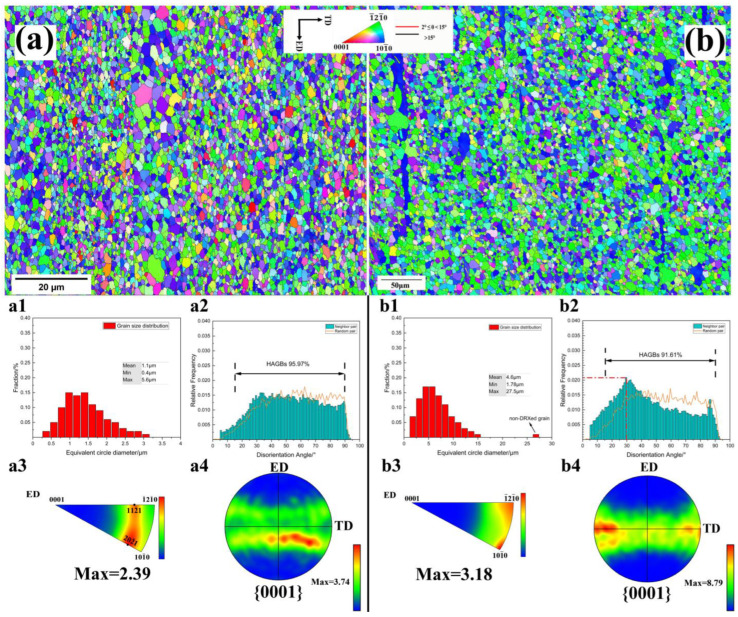
The EBSD analysis result of the as-RS (**a**,**a1**,**a2**,**a3**,**a4**) and as-homogenized (**b**,**b1**,**b2**,**b3**,**b4**) AZ31 hot-extruded rods.

Figure 6 shows the EBSD results of hot-extruded AZ31 rods in the ED. From Figure 6a,b, the grain size of as-RS AZ31 extruded rod and as-homogenized AZ31 extruded rod can be calculated more accurately. The equivalent circle diameter histograms of grain size (Figure 6a1,b1) were obtained by analyzing the statistics of about 70,000 grains; it can be seen that the average grain size of the as-RS AZ31 extruded rod was about 1.1 μm, with a minimum and maximum of 0.4 μm and 5.6 μm, respectively. In addition, the average grain size of as-homogenized AZ31 extruded rod is 4.6 μm, with a minimum grain size of 1.78 μm and a maximum grain size of 27.5 μm.

### 3.3. Texture Analysis of Hot-Extruded Rods

Figure 6a,b clearly show the grain orientation of as-RS and as-homogenized AZ31 extruded rod. Red and black grain boundaries represent low-angle grain boundaries (LAGBs, 2° ≤ θ < 15°) and high-angle grain boundaries (HAGBs, θ ≥ 15°), respectively. The quantitative statistical chart of the disorientation angle distribution (Figure 6a2,b2) shows the distinction between as-RS and as-homogenized AZ31 extruded rod. From the analysis of neighboring pairs and random pairs, it can be observed that the number fraction of both as-RS AZ31 extruded rods displays similar values at 30–90° (Figure 6a2), suggesting that these fine grains have more random orientations. However, Figure 6b2 shows a distinct peak at 30° in the neighboring pair analysis, indicating that the grain orientation of most adjacent grains in an as-homogenized AZ31 extruded rod is 30°.

Figure 6a3,b3 show the ED IPF that the texture component of as-RS AZ31 extruded rod is different from that of as-homogenized AZ31 extruded rod. As-RS AZ31 extruded rod constructs <$11\bar{2}1$>/<$20\bar{2}1$> double texture along the ED, while as-homogenized AZ31 extruded rod forms <$10\bar{1}0$>/<$\bar{1}2\bar{1}0$> double texture. Moreover, the texture component of as-homogenized AZ31 extruded rod has a higher intensity than that of as-RS AZ31 extruded rod. The lower intensity of the <$11\bar{2}1$>/<$20\bar{2}1$> texture component only reaches a peak value of 2.39, which has not been reported in the research on hot-extruded AZ31 rod.

Figure 6a4,b4 show the {0001} plane pole figure (PF) of as-RS and as-homogenized AZ31 extruded rod, showing that the polar density of both rods expands and forms a band on the TD, but the maximum intensity is 3.74 for as-RS AZ31 extruded rod and 8.79 for as-homogenized AZ31 extruded rod, respectively. Combined with the ED IPF (Figure 6a3,b3), it indicates that the as-RS AZ31 extruded rod has a much weaker texture without specific crystallographic directions parallel to the ED. The {0001} PF (Figure 6b4) suggests that the as-homogenized AZ31 extruded rod may have a prismatic texture parallel to the ED.

**Figure 7:**
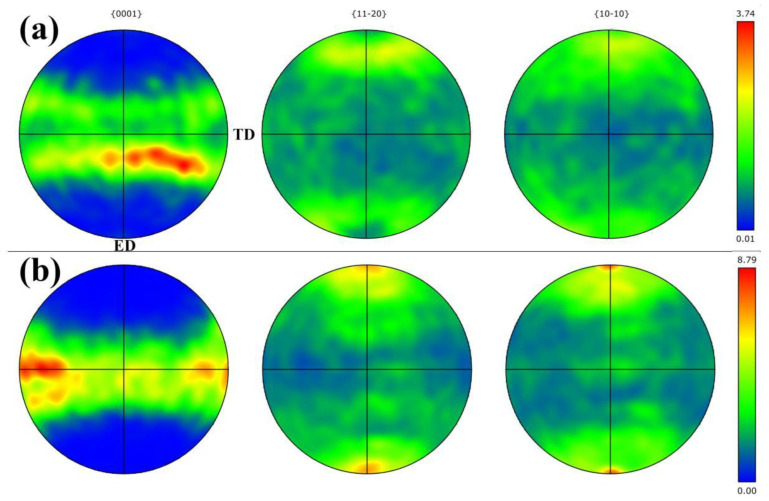
{0001}, {$11\bar{2}0$} and {$10\bar{1}0$} PF of as-RS (**a**) and as-homogenized (**b**) AZ31 hot-extruded rods.

Figure 7 shows the more PF results of the {0001}, {$11\bar{2}0$} and {$10\bar{1}0$} plane of as-RS and as-homogenized AZ31 extruded rod. Evidently, it can be seen that the as-homogenized AZ31 hot-extruded rod does have {$11\bar{2}0$} and {$10\bar{1}0$} prismatic texture (Figure 7b), whose grain’s prismatic plane is parallel to the ED. However, the polar density distribution of the as-RS AZ31 extruded rod is relatively uniform and low in these planes. Hence, based on the texture analysis, there is a non-fibrous component in the as-RS AZ31 extruded rod along the ED, whose grain orientation shows a more random result. According to Huppman et al. [17], the cast AZ31 extruded rod had <$10\bar{1}0$>/<$\bar{1}2\bar{1}0$> texture component when the extrusion temperature was between 250 °C and 300 °C. However, the as-RS AZ31 extruded rod shows a different result in terms of texture type (Figure 6a3).

**Figure 8:**
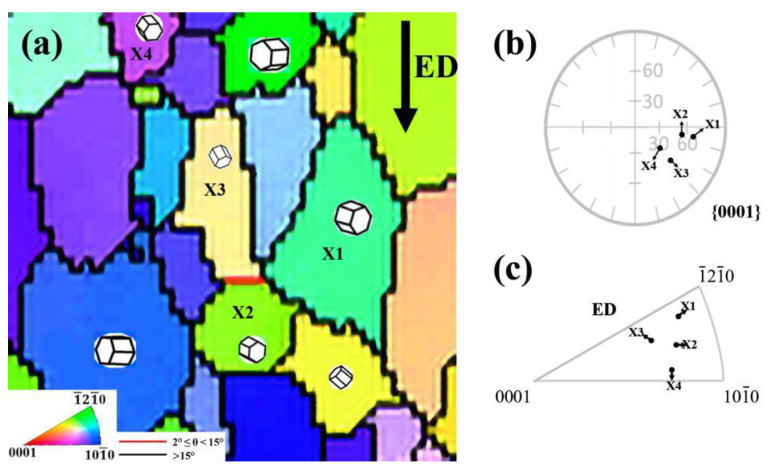
Specific crystallographic direction of the grains (X1, X2, X3, and X4) with <$11\bar{2}1$>/<$20\bar{2}1$ > texture component. (**a**) IPF map; (**b**) {0001} PF; (**c**) ED IPF.

Figure 8a shows some specific crystallographic directions of <$11\bar{2}1$>/<$20\bar{2}1$> texture components. The X1, X2, X3, and X4 are the grains of the as-RS AZ31 extruded rod selected in Figure 6a. The {0001}PF and ED IPF are shown in Figure 8b,c, respectively, indicating that the basal planes of the <$11\bar{2}1$>/<$20\bar{2}1$> texture component, such as X1, X2, X3, and X4 grains, are at an angle of 30~50° to the extrusion axis. Therefore, the crystallographic directions of X1, X2, X3, and X4 further demonstrate that most grains of as-RS AZ31 extruded rod are in random orientation.

On the whole, the main reason for the different texture type and low intensity is that the initial microstructure of as-RS AZ31 is completely distinct from that of as-homogenized AZ31. As discussed above, the grain size of the as-RS AZ31 is finer than that of the as-homogenized AZ31. Liu et al. [35] believed that grain refinement leads to high flow stress, which activates <c + a> dislocations to coordinate plastic deformation. Wei et al. [36] also concluded that the high flow stress in the fine-grained sample is believed to be the reason for the activation of non-basal slips. They found that with grain refinement in pure Mg from 125 to 5.5 μm, it could activate more non-basal dislocations. Meanwhile, the study showed that the initial microstructure has a strong influence on the hot working behavior of AZ31 alloy, which is due to the effect of the initial texture on the dynamic recrystallization (DRX) process [15,37]. Jiang et al. [38] found that the different recrystallization behaviors lead to the formation of various texture types during the extrusion of cast AZ31. They illustrated that the random orientation grains in Figure 8a were caused by discontinuous dynamic recrystallization (DDRX) and that continuous dynamic recrystallization (CDRX) was the way for the <$10\bar{1}0$> texture component turns into <$\bar{1}2\bar{1}0$> texture component. They suggested that the activation of non-basal slip dislocation is a guarantee to enable DDRX. At the same time, Zhang et al. [39] demonstrated that high stored strain energy facilitates DDRX in the sliding friction treatment of AZ31. Apparently, the initial fine RS microstructure can have both of these features. Besides, the other reason is the extrusion temperature. Cha et al. [11] concluded that the higher temperature (over 350 °C) would reduce the accumulated strain energy and thus weaken the DRX behavior during the extrusion of AZ31. Consequently, in this study, the finer initial microstructure of as-RS AZ31 not only can provide more non-basal slips but also can take advantage of generating stored strain energy. In addition, since at the relatively low extrusion temperatures (250 °C), there can be accumulated sufficient strain energy to fully generate DRX, resulting in an unusual <$11\bar{2}1$>/<$20\bar{2}1$> texture.

### 3.4. Mechanical Proprieties Hot-Extruded Rods

**Figure 9:**
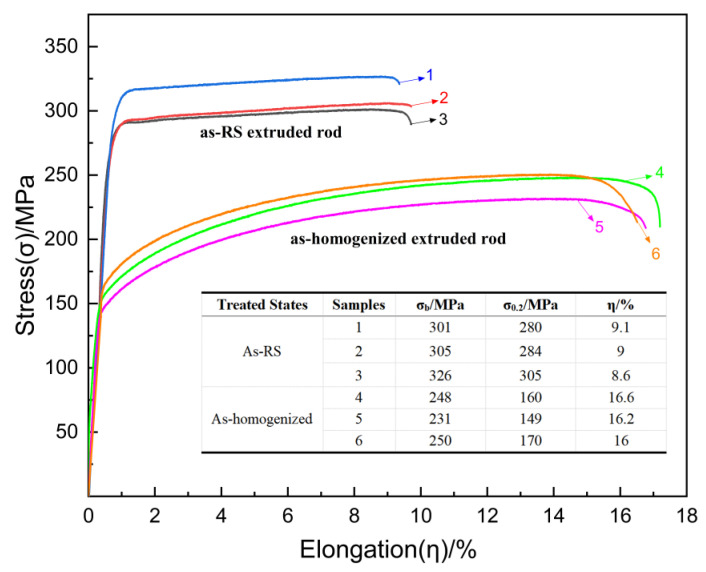
Engineering tensile stress-strain curves of as-homogenized and as-RS AZ31 extruded rods.

Figure 9 with the inserted table shows the engineering tensile stress-strain curves at room temperature of as-homogenized and as-RS AZ31 extruded rods. The average tensile strength (σ_b_), yield strength (σ_0.2_), and elongation of as-RS AZ31 extruded rod are 310.7 MPa, 289.6 MPa, and 8.9%, respectively. In addition, those of as-homogenized AZ31 extruded rod are 243.0 MPa, 159.7 MPa, and 16.3%, respectively. The tensile strength and yield strength of as-RS rod are increased by 27% and 81.3%, respectively, compared with as-homogenized rod.

According to the Hall-Petch relationship, σ_0.2 _= σ_0 _+ kd^−1/2^, it can be concluded from experiments and relevant literature data that the k value of conventional AZ31 extruded rod is 0.20 MPa/m^−1/2^ and the σ_0_ value is 85 MPa [40,41]. Therefore, the calculated result of the yield strength (σ_0.2_) of as-homogenized AZ31 extruded rod with an average grain size of 4.6 μm (Figure 6b1) is 178 MPa. In addition, that of as-RS AZ31 extruded rod with an average grain size of 1.1 μm (Figure 6a1) is 276 MPa. Compared with the average yield strengths shown in Figure 9, which are 159.7 MPa and 289.6 MPa, respectively, it can be assumed that the mechanical improvement of as-RS AZ31 extruded rod is mainly due to grain refinement strengthening.

Figure 9 also shows that the average elongation of the as-RS AZ31 extruded rod is 8.9%, which is much lower than that of the as-homogenized AZ31 extruded rod. In Figure 9, the samples 1, 2, and 3 both fracture during the process of work hardening, inducing low plasticity and indicating that the as-RS AZ31 extruded rod failed to coordinate plastic uniformity during the activation of new dislocation sources or the movement of existing dislocations. It is also well understood that the emergence, migration, and accumulation of dislocations govern plastic deformation [42]. Ashby proposed that geometrically necessary dislocations (GNDs) are generated rapidly and dominate the work-hardening processes for inhomogeneous deformation in polycrystals [43]. In addition, according to the references [44,45], uniform plastic deformation requires grain boundaries to coordinate the plastic incompatibilities between distinctly oriented grains defined by GNDs, and the low quantity of GND density is a consequence of the homogeneous deformation behavior of the polycrystalline ultrafine-grained structure.

**Figure 10:**
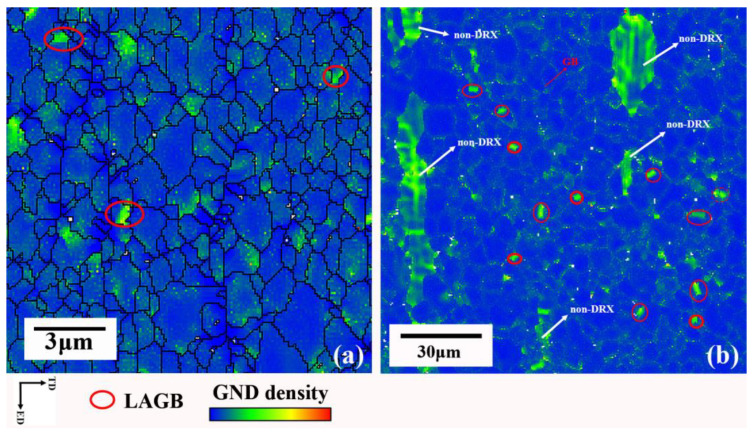
GND maps of (**a**) as-RS AZ31 extruded rod and (**b**) as-homogenized AZ31 extruded rod.

Figure 10 shows the GND maps detected by EBSD of as-RS AZ31 and as-homogenized AZ31 extruded rods at microscale. Except for the LAGBs and non-DRXed area, which naturally contain a high density of GNDs because GND density is strongly dependent on crystal orientation [46], it can be seen that the GND density of the as-RS AZ31 extruded rod is not significantly concentrated at the grain boundaries but randomly distributed in the DRXed grain interiors or part of the grain boundaries (Figure 9a). In contrast, that of the as-homogenized AZ31 extruded rod is highly concentrated at the grain boundary (Figure 9b), which validates that the as-RS AZ31 extruded rod with a ultrafine-grained structure undergoes a uniform deformation during the extrusion process.

By the way, the main reason for the lower plasticity in the as-RS AZ31 extruded rod is that it is possible that the powder oxidation of as-RS AZ31 was caused by the ball milling process. The unpredictable oxide composites may restrict the effect of GND between particles and even grains, resulting in worse coordinate ability in plastic deformation and lower elongation [47].

## 4. Conclusions

The present study illustrates the differences between rapid solidification and conventional solidification of AZ31 alloy during hot extrusion, which were investigated in detail by EBSD, TEM, mechanical tests, etc. The main results of this study are summarized as follows:

(1) Under the extrusion temperature of 250 °C and the extrusion speed of 6 m/min, the as-RS AZ31 extruded rod can achieve higher ultimate tensile strength (310 MPa) and yield strength (290 MPa) than those of the as-homogenized AZ31 extruded rod, which are increased by 27% and 81.3%, respectively; the latter is significantly improved.

(2) The as-RS AZ31 shows a finer microstructure than the as-homogenized AZ31 and has a non-fibrous texture, with the c-axis directions of most grains being about 30° to the thermal gradient. Furthermore, the average grain size of as-homogenized extruded rod is 4.6 μm, while that of as-RS extruded rod is only 1.1 μm, including fine sub-grains of about 200–400 nm and nanoparticles of about 10–30 nm.

(3) The as-RS AZ31 extruded rod shows a more random grain orientation, an unusual <$11\bar{2}1$>/<$20\bar{2}1$> component, and weak texture, which is different from the as-homogenized AZ31 extruded rod. The factors that contribute to this are the finer initial microstructure of as-RS AZ31 and the relatively low extrusion temperature.

(4) According to the calculation of the Hall-Petch relationship, the improvement in mechanical properties of the as-RS AZ31 extruded rod is mainly attributed to grain refinement, while the reduction in elongation is probably related to the powder oxidation during ball milling, which restricts the effect of GND between particles and even grains.

## Data Availability

Not applicable.
